# High Prevalence and Associated Risk Factors for Impaired Renal Function and Urinary Abnormalities in a Rural Adult Population from Southern China

**DOI:** 10.1371/journal.pone.0047100

**Published:** 2012-10-09

**Authors:** Qinghua Liu, Zhibin Li, Hui Wang, Xiaochao Chen, Xiuqing Dong, Haiping Mao, Jiaqing Tan, Ning Luo, Richard J. Johnson, Weiqing Chen, Xueqing Yu, Wei Chen

**Affiliations:** 1 Department of Nephrology, The First Affiliated Hospital, Sun Yat-sen University, Key Laboratory of Nephrology, Ministry of Health of China, Guangzhou, People’s Republic of China; 2 Epidemiology Research Unit, Translational Research Center, The First Affiliated Hospital, Sun Yat-sen University, Guangzhou, People’s Republic of China; 3 Department of Epidemiology and Preventive Medicine, School of Public Health of Guangzhou Medical University, Guangzhou, People’s Republic of China; 4 Department of Cardiology, The Fifth Affiliated Hospital, Sun Yat-sen University, Zhuhai, People’s Republic of China; 5 Division of Renal Diseases & Hypertension, University of Colorado Denver, Aurora, Colorado, United States of America; 6 Department of Epidemiology and Preventive Medicine, School of Public Health of Sun Yat-sen University, Guangzhou, People’s Republic of China; University of Sao Paulo Medical School, Brazil

## Abstract

**Background:**

The prevalence of chronic kidney disease (CKD) has increased and will continue to rise worldwide. However, data regarding the prevalence of CKD in a rural area of China are limited. We therefore investigated the prevalence and associated risk factors of impaired renal function and urinary abnormalities in an adult rural population in southern China.

**Methods:**

Between December 2006 and January 2007, residents older than 20 years from four villages in Zhuhai city were randomly selected using a stratified, multistage sampling technique. All participants were interviewed and tested for hematuria, albuminuria and estimated glomerular filtration rate (eGFR). The associations between age, gender, diabetes mellitus, hypertension, hyperuricemia, education level and indicators of renal damage were examined.

**Results:**

Overall, 1,214 subjects were enrolled in this study. After adjustment for age and gender, the prevalence of albuminuria was 7.1% (95% CI: 4.5, 8.1), reduced eGFR was 2.6% (95% CI: 1.7%, 3.3%), and hematuria was 4.6% (95% CI: 3.3%, 6.0%). Approximately 13.6% (95% CI: 12.0%, 15.1%) of the patients had at least one indicator of renal damage, but only 8.3% were previously aware. Age, diabetes, hyperlipidemia, hypertension, hyperuricemia, use of nephrotoxic medications, coronary heart disease and history of CKD were independently associated with impaired renal function and urinary abnormalities. Additionally, age, diabetes, and hypertension were independently associated with albuminuria. Age, hypertension, hyperuricemia, central obesity, and coronary heart disease were independently associated with reduced renal function.

**Conclusions:**

The high prevalence and low awareness of impaired renal function and urinary abnormalities in this population illustrates the urgent need to implement a CKD prevention program in the rural areas of southern China.

## Introduction

The global epidemic of chronic kidney disease (CKD) presents a challenge and a major public health problem for high-income developed countries, as well as developing countries, such as China [1]–[7]. CKD has worsened by the enormous disease burden of hypertension and the high incidence of diabetes in this population [8], [9]. China is the most populous country in the world, with great diversity. Overall, 57% of the total population resides in rural areas. We have previously reported that there is a high prevalence of CKD in the adults of Guangzhou city in Southern China, which has a relatively developed economy [10]. However, data regarding the rates of CKD in the rural areas of China remain unclear. We therefore assessed the prevalence and awareness of impaired renal function and urinary abnormalities in a rural area of southern China, and evaluated whether the frequency of impaired renal function and urinary abnormalities correlated with the prevalence of hypertension and diabetes as observed in urban populations.

## Results

Of the 1,214 subjects that were enrolled into the study, 1,186 had a complete data set and were entered into the final analysis. The demographic and clinical characteristics of the study population are shown in Table 1, in which male and female participants are compared. The age- and gender-adjusted prevalence of hypertension and diabetes were 22.0% and 4.5%, respectively. Notably, 6.2% of this population (no significant difference in terms of gender) used potentially nephrotoxic medications that included Chinese herbs, such as *Aristolochia* spp.

**Table 1:** Sociodemographic and clinical characteristics of the study population.

	Total	Male 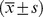	Female 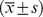	*P*-value
Age (year)	49.4±13.5	50.4±14.6	48.8±12.7	0.06
Weight (kg)	57.3±9.9	61.7±9.9	54.6±9.1	<0.001
Serum creatinine (µmol/L)	62.2±16.1	75.6±15.2	54.1±9.9	<0.001
Blood cholesterol (mmol/L)	5.6±1.2	5.4±1.2	5.7±1.2	<0.001
Serum triglyceride (mmol/L)	1.3±0.9	1.3±0.8	1.3±0.9	0.7
Blood uric acid (µmol/L)	325.3±96.1	370.9±95.4	297.7±85.7	<0.001
Fasting blood glucose (mmol/L)	5.6±1.5	5.6±1.7	5.6±1.5	0.8
Systolic blood pressure (mmHg)	128.6±21.3	129.8±19.9	127.9±22.1	0.1
Diastolic blood pressure (mmHg)	81.6±10.6	82.8±10.9	80.9±10.3	0.003
Body mass index (BMI)	23.0±3.3	22.9±3.2	23.1±3.4	0.3
≥High school education (%)	20.2	30.3	14.2	<0.001
Health insurance coverage (%)	75.5	76.5	75.5	0.16
Income (RMB/month)	1699.7	1825.5	1622.2	0.07
Smoker (%)	29.3	71.3	4.0	<0.001
History of CKD (%)	4.3	6.6	3.0	0.004
Family history of diabetes (%)	5.2	6.2	4.7	0.28
Family history of hypertension (%)	23.1	23.0	23.2	0.95
Family history of CKD (%)	3.7	5.2	3.1	0.08
Repeated respiratory tract infection (%)	9.3	12.0	7.7	0.02
Nephrotoxic medications (%)	6.2	6.6	5.9	0.64
Central obesity (%)	44.6	39.2	55.7	0.08
Coronary heart disease (%)	6.9	5.4	4.9	0.93
Stroke (%)	1.4	1.4	1.4	1.00
Cardiovascular diseases (%)	4.6	4.2	4.8	0.67
Hypertension (%)*	22.0	24.0	20.0	0.09
Diabetes (%)*	4.5	4.9	3.9	0.42
Hyperlipidemia (%)*	50.5	51.9	49.1	0.12
Hyperuricemia (%)*	15.4	25.3	5.4	<0.001
Metabolic syndrome (%)*	10.3	6.7	14.0	<0.001
eGFR<60 ml/min/1.73 m^2^ (%)*	2.9	3.1	2.2	0.07
ACR>30 mg/g (%)*	7.2	6.8	7.6	0.70
Hematuria (%)*	4.8	2.5	6.4	<0.001
Impaired renal function and/or urinary abnormalities (%)*	13.8	12.6	14.8	0.13

* Prevalence adjusted by age and gender distribution.

Abbreviations: ACR  =  urinary albumin-to-creatinine ratio; eGFR  =  estimated glomerular filtration rate.

There are 7 families in each of that 2 positive cases were detected in total 403 households in our survey. And among these 7 families, only two families have positive cases that were first degree relatives. The detailed information about these 7 families are shown in Table S1.

### Prevalence and Awareness of Indicators of Kidney Damage

#### Albuminuria

The adjusted prevalence of albuminuria was 7.1% (95% CI: 4.5%–8.1%, Table 2). Microalbuminuria and macroalbuminuria were detected in 5.9% (95% CI: 4.0%–7.8%) and 1.2% (95% CI: 0.6%–1.7%) of participants, respectively. The prevalence of albuminuria was similar between women and men (7.6% and 6.8%, respectively, *P*  = 0.70) and increased with advancing age in both genders (*P*<0.001 for both women and men across age groups). The prevalence of albuminuria was highest among subjects with diabetes or hypertension (17.5% and 13.1%, respectively). In subjects without hypertension or diabetes, the prevalence of albuminuria was only 5.6% and 3.3%, respectively. Of all the participants with albuminuria, only 6.8% was aware of the presence of albuminuria (5.3% and 7.6% in female and male participants, respectively). Individuals with diabetes, hypertension and cardiovascular diseases had relatively higher awareness levels (18%, 21% and 15%, respectively).

**Table 2:** Comparison of prevalence of adjusted indicators of kidney damage and related diseases between Doumen (a rural area of Zhuhai) and Guangzhou (urban data [10]).

Indicators		Prevalence inGuangzhou (%, age,gender adjusted)	95% CI(%)	Prevalence in Doumenof Zhuhai (%, age,gender adjusted)	95% CI (%)
**ACR**	**Albumiuria**	6.6	5.6∼7.6	7.1	4.5∼8.1
	**Microalbumiuria**	5.8	4.3∼6.2	5.9	4.0∼7.8
	**Macroalbumiria**	0.8	0.4∼0.8	1.2	0.6∼1.7
**Reduced eGFR**		3.2	2.8∼3.7	2.6	1.7∼3.3
**Hematuria**		3.8	3.4∼4.3	4.6	3.3∼6.0
impaired renal function and/or albuminuria		9.1	8.3∼9.8	10.0	8.1∼11.6
Impaired renal function and/or urinary abnormalities		12.1	11.3∼12.9	13.6	12.0∼15.1
**Hypertension**		19.2	18.2∼20.2	21.9	18.5∼24.2
**Diabetes**		5.5	4.9∼6.1	4.4	3.3∼5.7
**Hyperlipidemia**		41.4	40.1∼42.6	50.3	46.3∼51.3
**Hyperuricemia**		26.8	25.5∼27.8	15.1	12.3∼16.5
**Metabolic syndrome**		14.0	13.1∼14.9	10.1	8.3∼12.0

#### Decreased eGFR

The age- and gender-adjusted prevalence of eGFR <60 mL/min/1.73 m^2^ was 2.9% (95% CI: 1.8%, 3.6%) (Table 2) and increased with increasing age in both genders (*P*<0.001, respectively). The prevalence of eGFR <60 mL/min/1.73 m^2^ was highest among subjects with diabetes or hypertension (8.2% and 6.6%, respectively). In subjects without diabetes or hypertension, the prevalence was only 2.1% and 1.7%, respectively. The awareness of decreased eGFR was 12.8% among all the individuals with decreased eGFR, and was 26.5% and 22.0% in the groups of individuals with diabetes and hypertension, respectively.

#### Hematuria

Hematuria was present in 4.6% (95% CI 3.3%–6.0%) of participants. The prevalence of hematuria was significantly higher in women than men (6.4% and 2.5%, respectively, *P*<0.001). The awareness of albuminuria was 3.1% in this population.

### Prevalence and Awareness Rate of Impaired Renal Function and Urinary Abnormalities

The adjusted prevalence of impaired renal function and urinary abnormalities was 13.6% (95% CI: 12.0%–15.1). If the presence of hematuria was not included, the adjusted prevalence of impaired renal function and urinary abnormalities was 10.1% (95% CI: 8.1%–11.6%) (Table 2). The prevalence of impaired renal function and urinary abnormalities was similar in men and women (12.6% and 14.8%, respectively, *P*  = 0.13), and the increasing trend in prevalence of impaired renal function and urinary abnormalities with increasing age was observed between both genders (*P*<0.001 across age groups). The impaired renal function and urinary abnormalities prevalence was also greater among individuals with diabetes compared to those without diabetes (27.4% and 11.3%, respectively, *P*<0.001), cardiovascular disease than those without cardiovascular disease (35.3% and 11.9%, respectively, *P*<0.001), hypertension than those without hypertension (24.6% and 13.1%, respectively, *P*<0.001), and with metabolic syndrome than those without metabolic syndrome (21.7% and 15.9%, respectively *P*<0.001). According to age group, impaired renal function and urinary abnormalities was more prevalent among individuals aged >70 years (37.9%) than those aged 60–69 years (19.3%), 50–59 years (15.9%), 40–49 years (14.8%), 30–39 years (12.0%) or 20–29 years (9.9%). In terms of education level, impaired renal function and urinary abnormalities was more prevalent among people with less than a high school education (22.1%) compared to those with at least a high school education (15.7%, *P*<0.001).

The overall awareness rate was 8.3% in this population. Females with impaired renal function and urinary abnormalities had a lower awareness of their illness than their male counterparts (7.1% vs. 9.2%, respectively). Among participants with stage 1, stage 2, stage 3 and stage 4, the awareness rate of impaired renal function and urinary abnormalities was 4.8%, 6.7%, 10.5% and 42.9%, respectively. Subjects with either diabetes or hypertension had a lower awareness than subjects with both diabetes and hypertension (12.1%, 14.9% and 26.5%, respectively, *P*  = 0.02). Compared with participants without albuminuria or with microalbuminuria, subjects with macroalbuminuria had higher awareness rate (2.1%, 8.3% and 14.5%, respectively, *P*<0.001).

### Comparison of Impaired Renal Function and Urinary Abnormalities Prevalence and other Risk Factor Diseases Between Studies in Southern China

Table 2 also shows the age- and gender-adjusted prevalence of impaired renal function or urinary abnormalities, including hypertension, diabetes, hyperlipidemia, hyperuricemia and metabolic syndrome between the rural (Doumen Zhuhai) and urban areas (data were adopted from our previous study in Guangzhou [10]). The prevalence of impaired renal function or urinary abnormalities overall and of each indicator were similar between rural and urban populations in southern China. Compared with participants in the urban area, participants in the rural area had a lower prevalence of diabetes (rural vs. urban, 4.5% vs. 5.5%), metabolic syndrome (10.3% vs. 14.0%) and hyperuricemia (15.4% vs. 26.8%), while a higher prevalence of hypertension (22.0% vs. 19.2%) and hyperlipidemia (50.5% vs. 41.4%) was observed in the rural population.

### Associated Risk Factors of Impaired Renal Function and Urinary Abnormalities

The adjusted OR for the presence of impaired renal function and urinary abnormalities is shown in Table 3. Older age, diabetes, hyperlipidemia, hypertension (either high systolic or diastolic blood pressure), hyperuricemia, coronary heart disease, history of CKD and the usage of nephrotoxic medications were independently associated with the occurrence of impaired renal function and urinary abnormalities. A higher level of education (at least a high school education) was a protective factor against impaired renal function and urinary abnormalities. When all other variables were adjusted, older age, diabetes and hypertension (either high systolic or diastolic blood pressure) were independently associated with albuminuria. Furthermore, older age, hypertension, hyperuricemia, central obesity and coronary heart disease were independently associated with reduced renal function.

**Table 3:** Adjusted OR for the presence of impaired renal function and/or urinary abnormalities, albuminuria or impaired renal function.

Variable	P-value	Adjusted OR (95% CI)
**Impaired renal function and/or urinary abnormalities**		
Age (↑ 10 year)	0.03	1.19 (1.06∼1.35)
Diabetes	0.04	1.70 (1.03∼2.9)
Hyperlipidemia	0.02	2.13 (1.41∼5.22)
Hypertension	0.01	1.63 (1.12∼2.28)
High systolic BP	0.001	1.89 (1.29∼2.67)
High diastolic BP	0.03	1.51 (1.04∼2.16)
Hyperuricemia	0.04	1.53 (1.05–2.25)
Nephrotoxic medications	0.02	2.03 (1.16∼3.53)
Coronary heart disease	0.02	2.41 (1.11∼5.27)
History of CKD	0.004	2.70 (1.39∼5.49)
Education	0.01	0.85 (0.71∼0.90)
**Albuminuria**		
Age (↑ 10 year)	0.03	1.33 (1.09∼1.62)
Diabetes	0.003	2.72 (1.48∼5.50)
Hypertension	<0.001	3.11 (1.70∼5.29)
High systolic BP	<0.001	3.21 (2.21∼6.09)
High diastolic BP	<0.001	3.28 (1.99∼5.48)
**Impaired renal function**		
Age (↑ 10 year)	<0.001	2.69 (1.92∼3.82)
Hypertension	0.01	1.93 (1.25∼2.31)
Central obesity	0.03	1.33 (1.10∼2.51)
Coronary heart disease	0.008	4.28 (1.40∼6.60)
Hyperuricemia	0.01	2.31 (1.80∼4.11)

## Discussion

The present study was a population-based survey of adult individuals from a southern China rural area, which assessed the prevalence of impaired renal function and urinary abnormalities. Findings from the present study indicate that approximately 13.8% of adults in this population had one or more indicators of kidney damage. Of the patients diagnosed with albuminuria, hematuria and reduced eGFR, only 8.3% were aware of their condition. Compared with our previous urban study in Guangzhou city of Southern China [10], the rural area population appeared to have a similarly high prevalence of impaired renal function and urinary abnormalities, but a lower awareness level of their disease.

These data highlight the increasing major health problem of kidney damage and the challenge of other CKD-related diseases in rural populations of southern China. The epidemic of CKD in recent years has been paralleled by similar trends in other risk factors for chronic disease, such as hypertension and diabetes. Data from several national surveys have also shown that hypertension and diabetes have reached epidemic proportions in the general adult population, which were not restricted to particular subgroups of the Chinese population [8], [9]. Notably, the increasing economic development and rapid urbanization in coastal rural areas such, as Zhuhai city in southern China, has led to dramatic increases in unhealthy lifestyles and behaviors, resulting in a sharp rise in CKD-related risk factors [8], [9]. Our study demonstrated that the rural population had a lower prevalence of diabetes, metabolic syndrome and hyperuricemia than an urban population in southern China, but surprisingly, the rural population had an even higher prevalence in hypertension and hyperlipidemia. Additionally, the proportion of individuals who were aware of their impaired renal function or urinary abnormalities in this study was exceedingly low, which may be partly related to the relatively low levels of education, and could also be attributed to the disadvantaged nature of this population in accessing healthcare services. Therefore, multi-disciplinary prevention strategies for rural China are urgently required to tackle the increase in CKD and related issues.

Our present findings for the overall prevalence of impaired renal function and urinary abnormalities are similar with those reported by the Handan Eye Study, which was conducted in adults >30 years of age in a northern rural Chinese population. That study reported that approximately 15.2% of adults had CKD (as defined by eGFR <60 ml/min/1.73 m^2^ or albuminuria) [11]. Interestingly, there was a remarkable discrepancy between the prevalence of kidney damage indicators between these two studies, even though the overall reported CKD prevalence was similar to that of impaired renal function and urinary abnormalities in our study.

In the Handan Eye Study, albuminuria was detected in 16.8% of participants and the age-standardized prevalence of albuminuria was 14.9% [11], which was substantially higher than the adjusted prevalence of albuminuria (7.2%) observed in the present study. This difference is likely due to multiple causes that include fundamental geographic differences between the northern and southern population of China. For example, the prevalence of hypertension was much lower in the present study (22.0%) than in the Handan Eye Study (40.5%). According to the prevalence of hypertension data recorded by the China National Nutrition and Health Survey 2002, the northern region of China had a much higher prevalence of hypertension compared with the central southern region of the country (27% and 17%, respectively) [8]. Furthermore, several other epidemiological studies in the rural area of northern China showed that 36.2%–44.1% of the rural adult population had hypertension [12]–[14]. Lifestyle differences may also play an important role in this regard. Of note, the northern Chinese have, on average, a higher dietary intake of sodium, a higher prevalence of overweight and obese individuals, greater alcohol intakes, and lower levels of physical activity compared with the southern regions of China [15]–[17]. All of these risk factors could account for the difference in prevalence of not only hypertension, but also impaired renal function and urinary abnormalities. Some of the differences may have been due to methodological challenges, such as creatinine measurement and calibration techniques. In the present study, the eGFR was calculated from the MDRD equation modified for Chinese CKD patients, and the serum creatinine was calibrated.

Unlike developed countries, in which the major causes of end-stage renal disease (ESRD) are diabetes mellitus and hypertension, the leading cause of ESRD in China remains glomerulonephritis, which accounts for 49.9% of all kidney diseases. Thereby, we also included hematuria as an indicator for kidney damage in our study.

In the rural population, our current work confirmed that regular usage of nephrotoxic medications (which included NSAIDs and Chinese herbal medicines containing aristolochic acid [18]) was a risk factor for impaired renal function and urinary abnormalities in Chinese individuals, which might assist in future prevention strategies for CKD in China. This finding is consistent with our observations from urban areas and the studies of other groups in China [5], [10], [19]–[21]. Herbal medications are widely used in China. They are more popular among the rural populations than the urban population because the healthcare system is relatively difficult to reach and herbal medicines are more easily available in the rural areas. We found a higher prevalence (6.2%) of the rural population using potentially nephrotoxic medications compared to the urban population in southern China (4.9%) [10]. Given these considerations, public education will be important, especially in rural areas, to counteract the commonly held view that herbal medicines are safer than modern medicines.

The current study had several limitations. First, the data was taken from a cross-sectional survey with a limited number of participants, which prevented the determination of whether risk factors were caused by or resulted from impaired renal function and urinary abnormalities. Second, only one urine sample examination was obtained per participant, which made it impossible to confirm whether the hematuria or albuminuria were persistent. As such, we use the term of impaired renal function and urinary abnormalities instead of CKD. Despite these limitations, we used a stratified, multistage sampling method to obtain a representative sample of the rural area of Zhuhai adult population with a high response rate. Additional strengths of the study include the fact that eGFR was calculated from the MDRD equation that was modified for data from Chinese CKD patients.

In conclusion, impaired renal function and urinary abnormalities is very common in this rural adult population of southern China, with disappointingly low rates of disease awareness. Our data underscore that comprehensive programs and concerted efforts need to be implemented to improve the prevention and detection of CKD and its risk factors in the rural population of southern China.

## Methods

### Ethics Statement

All participants gave their written informed consent prior to data collection. Illiterate participants had the information leaflet read out to them and provided a thumb impression. The Human Ethics Committees of The First Affiliated Hospital of Sun Yat-sen University, Guangzhou, China, approved the study.

### Study Population

This study was a cross-sectional study of impaired renal function and urinary abnormalities and its associated risk factors in a representative sample of the general adult population of the Zhuhai rural area in southern China between December 2006 and January 2007. The study samples were local residents aged 20 years or older who had lived in the region for 5 years or longer. Samples of participants were selected using a stratified, multistage sampling method. First, the district of Doumen was non-randomly selected from the two rural districts in Zhuhai city. In the second stage, two counties (Doumen and Jingan) were randomly selected from the five counties in the Doumen district. Then, four villages were randomly selected from these two counties. In the fourth stage, a simple random sampling method (random numbers generated by SPSS software) was used to select 100 households from each of the selected villages. In the final stage of the sampling, all subjects that fulfilled the inclusion criteria in the selected households were selected and stratified by age distribution based on the 2005 China Population Census Data. Altogether, 1,214 residents were selected from the four villages and enrolled into the study. Of these subjects, 1,186 completed the entire survey, giving a response rate of 97.7%.

### Screening Protocol and Evaluation Criteria

All researchers that participated in this study, including physicians and medical students, received intensive training on the standard screening protocol. Data were collected at the local village health centers. Each subject completed a questionnaire that documented the sociodemographic status of participants (e.g., age, gender, income and education level), personal and family health history (e.g., hypertension, diabetes and kidney disease), and lifestyle behavior (e.g., smoking, alcohol). Any history of medicinal usage with the potential for nephrotoxicity, including non-steroidal anti-inflammatories and herbs that contained aristolochic acid, was also recorded.

Anthropometric measurements were obtained using standard protocols and techniques described in previous studies [10], [22]. After shoes and heavy clothing were removed, each subject underwent body weight and height measurements using a calibrated scale. Venous blood was collected after an overnight fast of at least 10 h for the measurement of various biomarkers. A clean-catch, midstream, morning urine specimen was collected for the measurement of urinary albumin, creatinine and dipstick testing for the rapid screening of hematuria, which was then confirmed by microscopic analysis. All blood and urine samples were tested in the laboratories of The First Affiliated Hospital at Sun Yat-sen University.

### Albuminuria

Urinary albumin and creatinine levels were measured from a morning urine sample using an automatic analyzer (COBAS INTEGRA 400 plus, Roche, Basel, Switzerland). Creatinine was measured by Jaffe’s kinetic method and albumin by the immunoturbidimetic method. The urinary albumin-to-creatinine ratio (ACR, mg/g) was then calculated. Microalbuminuria and macroalbuminuria were defined as an increase in ACR between 30 and 299 mg/g and 300 mg/g or greater, respectively, according to the guidelines of the American Diabetes Association. The term “albuminuria” is used to describe the presence of either microalbuminuria or macroalbuminuria.

### Estimated Glomerular Filtration Rate (eGFR)

Blood was collected by venipuncture after overnight fasting. Serum creatinine (Scr) was measured using the same method to determine urinary creatinine. eGFR was calculated using the Modification of Diet in Renal Disease (MDRD) study equation modified for Chinese CKD patients [23]. Before the study, the laboratory in our center calibrated serum creatinine measurements with samples at the laboratory of the Peking University First Hospital, where the modified equation was developed [23]. The resulting calibration equation was as follows (R^2^ = 0.999): Calibrated Scr (mg/dl) = 0.893× Scr (mg/dl) +0.39.

To ensure quality control measures in the present study, creatinine was measured in 40 serum samples with a creatinine range of 0.5 to 15 mg/dl both at our laboratory and at the central laboratory of Peking University First Hospital. Reduced renal function was defined as an eGFR less than 60 mL/min/1.73 m^2^, where eGFR (ml/min/1.73 m^2^) = 175×Calibrated Scr (mg/dL)^−1.234^×age (year)^−0.179^ [female×0.79].

### Hematuria

Dipstick testing (Roche Diagnostics, Mannheim, Germany) was performed on morning spot urine samples. Subjects with hematuria of 1+ or greater were confirmed by microscopic analysis within 2 h of the dipstick. Urine samples were centrifuged at 1,500 *g* for 5 min, the supernatant was removed, and the sediment in the remaining supernatant was re-suspended. An aliquot (20 µl) of the sample was placed on a clean glass slide and examined using subdued bright-field illumination at magnifications of ×100 and ×400 under light microscopy. Three or more red blood cells per high-power field by microscopy were considered abnormal. Women who were actively menstruating were excluded from the urine test.

### Hypertension Status

Arterial blood pressure was measured using a mercury sphygmomanometer after subjects had rested for at least 15 min. The procedure for measuring blood pressure was according to the Joint National Committee VII criteria (JNC VII) [24]. Three readings were taken at 1 min intervals. The mean of these measurements was calculated, unless the difference between readings was greater than 10 mmHg, in which the mean of the two closest of the three readings was used. Hypertension was defined as systolic blood pressure (BP) ≥140 mmHg or diastolic BP≥90 mmHg, a self-reported diagnosis of hypertension, or the patient used anti-hypertensive medication in the last 2 weeks irrespective of blood pressure.

### Diabetic Status

Fasting blood glucose was measured enzymatically with a glucose oxidase method (COBAS INTEGRA 400 plus). Diabetes was defined as the use of insulin or oral hypoglycemic agents, a fasting plasma glucose ≥7.0 mmol/L (126 mg/dL), and/or a 2 h postprandial plasma glucose ≥11.1 mmol/L (200 mg/dL).

### Other Measurements

Serum total cholesterol, low-density lipoprotein (LDL)-cholesterol, high-density lipoprotein (HDL)–cholesterol, triglycerides and uric acid were measured by an auto analyzer (COBAS INTEGRA 400 plus). Hyperuricemia was defined as a serum uric acid level greater than 7.0 mg/dl. Cardiovascular disease, which was diagnosed by a physician, was defined as congestive heart failure, coronary heart disease, angina, stroke, or heart attack. Body mass index (BMI) was calculated as weight (in kilograms) divided by height squared (in square meters). Obesity was defined as a BMI >28 [25], and central obesity was defined as a waist measurement >90 cm for men or >80 cm for women [26]. Metabolic syndrome was defined according to the International Diabetes Federation (IDF) definition: the presentation of central obesity plus any two of the following risk factors: triglyceride concentration ≥1.70 mmol/l (150 mg/dl) or specific treatment for this lipid abnormality; HDL-cholesterol concentration <1.04 mmol/l (40 mg/dL) in men or <1.30 mmol/l (50 mg/dL) in women or a specific treatment for this lipid abnormality; BP≥130/85 mmHg or previously diagnosed hypertension currently under treatment; fasting plasma glucose ≥5.55 mmol/l (100 mg/dL); or previously diagnosed type 2 diabetes (http://www.pitt.edu/~super1/Metabolic/IDF1.pdf). CKD awareness was assessed using the question in the questionnaire: “Have you ever been told by a physician or healthcare professional that you had any kind of renal disease (glomerulonephritis), an abnormality in urine testing (hematuria or proteinuria), or an abnormal renal function test result?”.

### Statistical Analysis

Data entry and management were performed using Epidata software, version 3.0 (Epidata Association, Odense, Denmark). We conducted statistical analyses using SAS software (version 8.02; SAS Institute Inc., Cary, NC) and SPSS software, version 10.0 (SPSS, Inc., Chicago, IL USA). Data are presented as means ± standard deviation for continuous variables and as proportions for categorical variables. Quantitative variables were summarized as means and 95% confidence intervals (CI). Differences between subjects were analyzed by two-tailed unpaired Student’s *t*-tests for continuous data and by χ^2^ tests for categorical data. Univariate and multivariate logistic regression analyses were used to estimate odds ratios (OR) and CIs by comparing associated risk factors with impaired renal function and urinary abnormalities occurrence. To account for the clustering of correlation among individuals from the same household, prevalence odds ratios (POR) and 95% confidence intervals (95% CI) were calculated using logistic regression (PROC GENMOD) with generalized estimating equa tions (GEE). *P*-values <0.05 were considered to be significant.

The overall impaired renal function and urinary abnormalities prevalence, defined as the presence of albuminuria, hematuria or eGFR values <60 (ml/min/1.73 m^2^), was calculated and tested for interactions with demographic characteristics (i.e., age group, gender, and education level, smoking, and family history) and with CKD risk factors (diabetes, cardiovascular disease, hypertension, and body mass index). Estimates of demographic characteristics and risk factors were standardized according to age and gender in the Zhuhai standard population aged >20 years (data from the China Population Census in 2005; http://www.stats-zh.gov.cn/index.htm).

## Supporting Information

Table S1 Information of positive cases among same households.
(DOC)
