# Supplementary material for: High Prevalence and Associated Risk Factors for Impaired Renal Function and Urinary Abnormalities in a Rural Adult Population from Southern China
Source: PLoS One. 2012 Oct 9;7(10):e47100. doi: 10.1371/journal.pone.0047100 (PMC3467213; doi:10.1371/journal.pone.0047100)
Supplement: Table S1 — Information of positive cases among same households. (DOC) [file pone.0047100.s001.doc]

**Table S1:** Information of positive cases among same households

|  | Number of positive cases | Relationship |  |
| --- | --- | --- | --- |
| Family 1 | 2 | Spouse | Case 1: CKD stage 1, with hematuria and albuminuria;  Case 2: CKD stage 1, with hematuria |
| Family 2 | 2 | Spouse | Case 1: CKD stage 2, with hematuria;  Case 2: CKD stage 2, with albuminuria |
| Family 3 | 2 | Spouse | Case 1: CKD stage 1, with albuminuria;  Case 2: CKD stage 1, with hematuria |
| Family 4 | 2 | Sibling  (brother and sister) | Case 1: CKD stage 1, with hematuria;  Case 2: CKD stage 1, with albuminuria |
| Family 5 | 2 | Spouse | Case 1: CKD stage 2, with hematuria;  Case 2: CKD stage 3, with hematuria and albuminuria |
| Family 6 | 2 | Mother and daughter | Case 1: CKD stage 1, with hematuria;  Case 2: CKD stage 2, with albuminuria |
| Family 7 | 2 | Spouse | Case 1: CKD stage 1, with albuminuria;  Case 2: CKD stage 1, with albuminuria |
